# Understanding the Polymorphism of Cobalt Nanoparticles Formed in Electrodeposition—An In Situ XRD Study

**DOI:** 10.1021/acsmaterialslett.2c00861

**Published:** 2023-03-06

**Authors:** Xuetian Ma, Yifan Ma, Adelaide M. Nolan, Jianming Bai, Wenqian Xu, Yifei Mo, Hailong Chen

**Affiliations:** † Georgia Institute of Technology, the Woodruff School of Mechanical Engineering, 771 Ferst Drive, Atlanta, Georgia 30332, United States; ‡ University of Maryland, Department of Materials Science and Engineering, 4418 Stadium Drive, College Park, Maryland 20742, United States; § National Synchrotron Light Source II, Brookhaven National Laboratory, Upton, New York 11973, United States; ∥ Advanced Photon Source, Argonne National Laboratory, 9700 Cass Ave, Lemont, Illinois 60439, United States

## Abstract

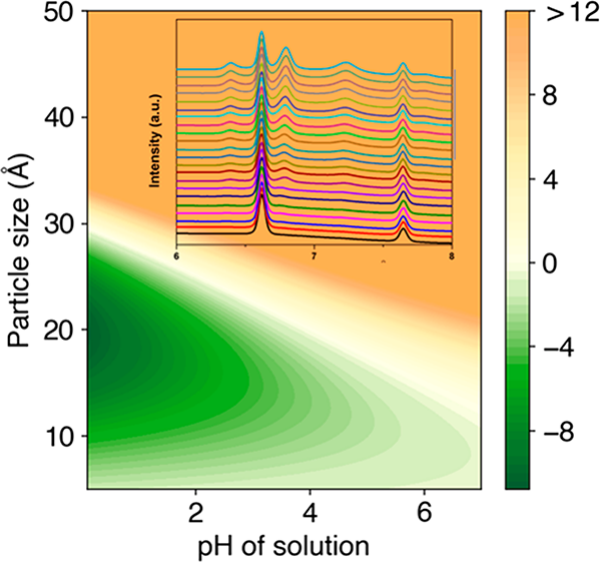

An advanced synchrotron-based *in situ* X-ray diffraction (XRD) technique is successfully developed and employed to track and monitor the formation and phase selection of cobalt (Co) in electrodeposition in real time and verify DFT computational results. The impacts of a number of controlling factors including the pH of the electrolyte and deposition overpotential are systematically studied. The results show that the yielded phase of the electrodeposited Co is controlled by both thermodynamics and kinetics. The low pH low overpotential condition favors the formation of the thermodynamically stable *fcc* phase. While the high pH high overpotential condition promotes the formation of the metastable *hcp* phase. The experimental results agree well with the nanometric phase diagram computed with DFT. Layer-by-layer alternative stacking of *fcc-hcp* polymorphic phases can be facilely fabricated by just varying the overpotential. This work not only offers an effective means to control the phase of electroplating of Co but also presents a new approach to reveal the fundamental insights of the formation of metals under electrochemical reduction driving force.

The long-standing challenge in materials engineering is to understand the formation process of materials and then apply controls to tune the crystal structure, morphology, and defects, etc., in order to achieve desired functionalities. This is particularly important yet difficult for nanomaterials, as they often form in off-equilibrium conditions and their formation process is poorly understood and is hard to predict with conventional phase diagrams. In a previous work,^1^ we established a nanometric phase diagram based on first-principles computations, which allows for quantitative understanding of the thermodynamics of nanoparticles of metallic cobalt (Co) and prediction of their stable polymorphic phases formed in solvothermal reduction reactions. Experimental results, including *in situ* XRD observation on the nucleation process, demonstrated that this computed nanometric phase diagram is very accurate and suggested that this approach may be applied to more materials and more nucleation conditions. It is then interesting and critical to further examine the effectiveness of this nanometric phase diagram under other formation conditions. In this work, we report our recent findings and new insights of the formation of metal under electrodeposition conditions and the phase selection rules, still with using Co as the model material.

Co is known to have two common polymorphs, the hexagonal close-packed (*hcp*) phase and the face-centered cubic (*fcc*) phase. In the bulk phase diagram,^2^*hcp* Co is the thermodynamically stable phase at lower temperatures, while the *fcc* phase is more stable above 450 °C.^2^ Nanoparticles of Co in different polymorphs showed different properties than that in magnetic, electrical, and catalytic applications.^3,4^*hcp* Co has higher coercivity and stronger magnetic anisotropy than *fcc* Co, making it suitable for magnetic recording and permanent magnet applications.^5^ On the other hand, the relatively magnetically soft *fcc* Co is more widely used in soft magnetic applications, such as power electronics and magnetic write heads.^6^ Many methods have been used to synthesize Co in different polymorphs at different length scales,^7−9^ including solvothermal reduction used in our previous work.^1^ In this work, we investigate Co synthesized in electrodeposition. Electrodeposition is widely used for the synthesis/manufacture of metals, alloys, polymers, and ceramics. It is low cost,^10^ scalable for mass production,^11^ capable of producing uniform and dense films on complex surfaces within short time.^12^ Most importantly, from physical chemistry point of view, electrodeposition is different than the chemical synthesis methods in that the driving force of the reaction is continuously tunable (i.e., the electrochemical potential can be conveniently controlled by the applied voltage), while in chemical reactions, the driving force is the chemical potential set by the reagents used, which oftentimes is not easy to tune in a wide range. Electrodeposition also offers more quantitative controls on the reaction and has more tuning factors,^13^ such as voltage, current density, temperature, concentration of electrolytes, etc., which is helpful to design systematic experiments to reveal the insights of the reactions. In addition, electrodeposited Co is also important for magnetic thin film applications.^14^ Both *fcc*(15) and *hcp*(16) Co have been reported to form under different electrodeposition conditions. However, a systematic understanding on why *fcc* and *hcp* Co phases form selectively and how the deposition parameters govern this process remains unclear, which warrants an in-depth investigation to *in situ* characterize the nucleation process. This is a significant challenge, not only because the size of the nuclei is very small but also because the deposition process takes place very fast at millisecond scale. With our effort, a platform that allows for *in situ* XRD characterization for electrodeposition has been built and successfully tested at multiple synchrotron beamlines. With using this platform, systematic electrodeposition experiments of Co were conducted and compared with chemical reduction experiments. The relationship between the nucleation process and the deposition conditions is discussed.

## Experimental Methods

The electrolyte for cobalt electrodeposition was composed of 0.5 M cobalt sulfate pentahydrate (99.9%, GFS Chemicals) in 20 mL of deionized water. Additives including 0.1 M boric acid (99.5%, BDH Chemicals) and 0.1 M sulfate acid (95–98%, BDH Chemicals) were used to tune and control the pH, for the examination of effects of pH on phase selectivity of Co. The chemical compositions of electrolyte used are listed in Table S1.

A two-electrode system was used for the potentiostatic electrodeposition processes with using a battery cycler (Arbin, BT2043). Copper foil with thickness of 0.005 in. and area of 2.5 cm^2^ was used as the working electrode, while platinum foil with an area of 0.25 cm^2^ was used as the counter electrode. The current density was controlled between 6 to 65 mA/cm^2^ for the working electrode to investigate the effect of overpotential on phase selectivity.

*Ex situ* XRD measurement was performed with using a D8 Advance X-ray Diffractometer (Bruker AXS) with a molybdenum radiation (λ Kα_1_ = 0.7093 Å). An *in situ* cell was designed to perform the *in situ* XRD observation at the synchrotron sources, as schematically shown in Figure S2. Each of the samples was deposited for 20 min to form a homogeneous Co film with thickness around 25–30 μm to avoid topotaxial growth induced by the substrate.^12^*In situ* synchrotron XRD was conducted at beamline 28-ID-2 at the National Synchrotron Light Source II (NSLS II) at Brookhaven National Laboratory and beamline 17-BM-B at the Advanced Photon Source (APS) at Argonne National Laboratory. The microstructure of Co nanoclusters at the early stage, which were obtained after 1 min deposition, was observed with using a scanning electron microscope (SEM) (Hitachi SU8010). All the electrochemical deposition and XRD measurements were conducted at room temperature (25 °C).

In a previous study on the solvothermal reduction of Co^1^, it was identified that the change of surface energy can drastically alter the total energy of the subnanometer crystalline nucleus, thus determining which phase would form in solution. The surface energy is significantly affected by both the size of the nucleus and the surface capping status, which can be tuned by pH or the concentration of surfactants. Under neutral or slightly basic pH (7–9), the size-impacted surface energy results a lower energy for *fcc* Co, which is actually the metastable phase in bulk, and makes it the dominant phase in the final product. Under higher pH (>14), the total energy of *hcp* Co is much lower than that of *fcc* Co, due to the capping of OH^–^ onto the surface, therefore resulting the dominant formation of *hcp* Co. In electrodeposition, the nucleation of Co takes place at the interface of the electrode and the electrolyte. The capping of ions and ligands in the electrolyte is still expected to have pronounced impact on the surface energy and thus the total energy of the nuclei, due to large area of particle-solution interface. Therefore, it is worthwhile to first examine the impact of the OH^–^ capping with tuning the pH. Second, to investigate the impacts of reaction driving force and kinetics to the phase selectivity, the overpotential of the electrochemical reduction was also tuned. A series of samples were prepared in electrolytes with various pH (0–1, 4–5, and 6) and with using different voltages (0.8 or1.5 V). The pH of the solution was controlled by adding boric acid and diluted sulfate acid. Overpotential is controlled by adjusting the voltage applied between the two electrodes. Because relatively high overpotential was used, hydrogen evolution can not be avoided. The reactions at the working electrode are
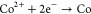
1
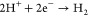
2The reaction at the counter electrode is
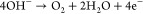
3

**Figure 1:**
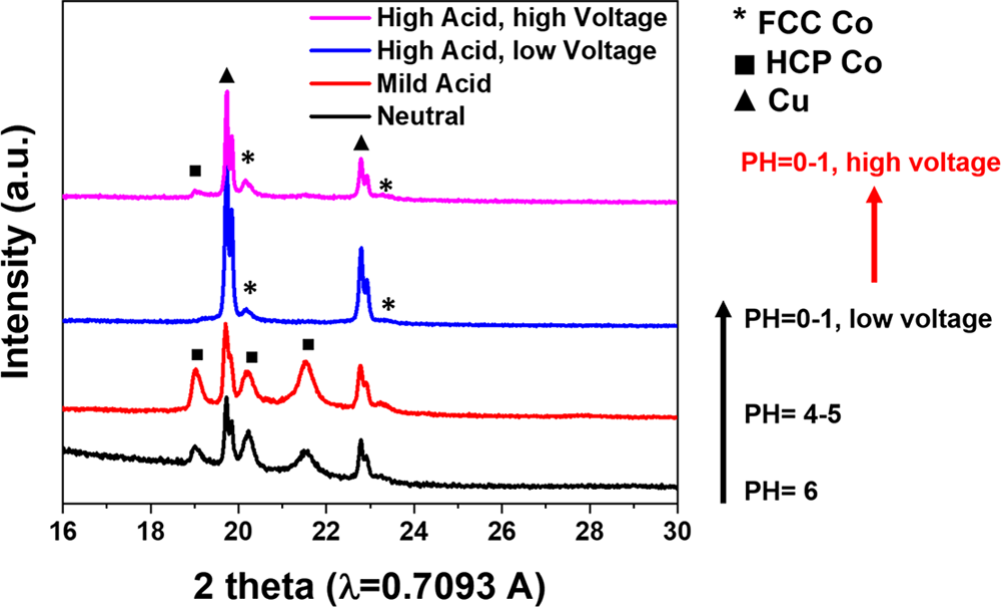
*Ex situ* XRD pattern of N, MA, HA-LV, and HA-HV samples under various pH conditions and different overpotential. The fcc phase, hcp phase, and Cu substrate are labeled with asterisk, square, and triangle, respectively.

**Figure 2:**
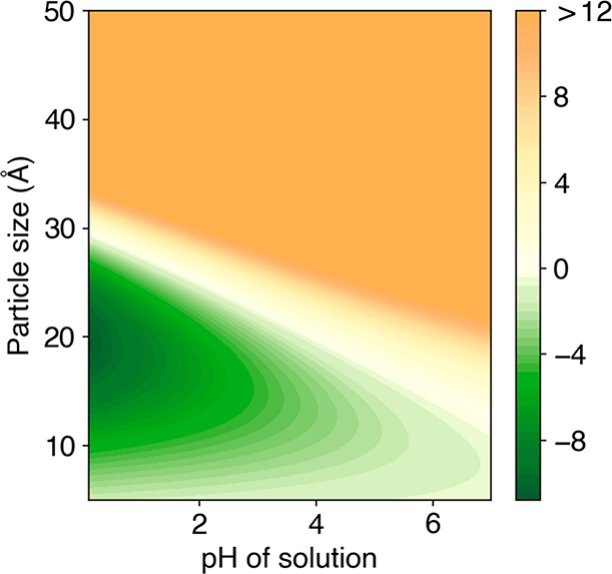
Energy difference of *fcc* Co with *hcp* Co with respect to varying particle sizes and pH level of the solution. The color bar shows the energy differences (*E*_fcc_ – *E*_hcp_) between the nanoparticles of the two phases.

Figure 1 shows the *ex situ* X-ray diffraction (XRD) patterns of the samples obtained after 20 min of deposition. The samples obtained in different electrolytes and under different overpotentials are denoted as N (neutral electrolyte), MA (mildly acidic electrolyte), HA-LV, (highly acidic electrolyte and low voltage), and HA-HV (highly acidic electrolyte and high voltage), respectively. For N, MA and HA-LV samples, the overpotential was controlled constant at 0.8 V vs an Ag/AgCl reference electrode, which is a condition close to equilibrium with low driving force. The deposited phases show pronounced dependence on the pH. There is a clear trend that relatively high pH favors the formation of *hcp* Co, while very low pH favors the formation of *fcc* Co. (The low intensity of *fcc* Co in sample HA-LV might be due to the smaller thickness of the deposited film, which was resulted from the lower current density, as shown in Table S1) This trend is the same as what was observed in the solvothermal synthesis.^1^ It is worth noting that, in previous solvothermal experiments, the acidic pHs were not able to be tested (because hydrogen formation would dominant in solvothermal if pH is below 7). Now with electrochemical deposition, acidic pH conditions can be tested. We conducted DFT computation with greater range of nucleation size and solution parameters representative to the electrodeposition condition and the result is plotted in Figure 2. It can be seen that the overall energy of Co nucleus is lower for *fcc* than *hcp* in low pH, agreeing with the trend observed in the experiments.

**Figure 3:**
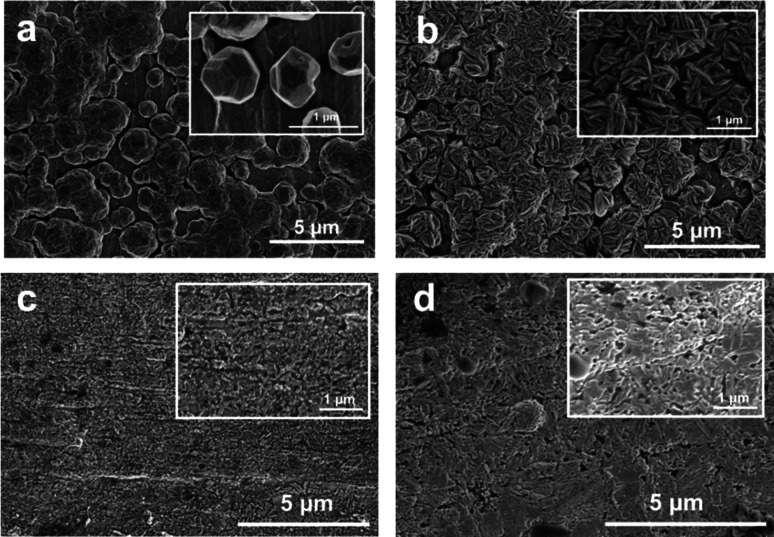
SEM images of N sample (a), MA sample (b), HA-LV sample (c), and HA-HV sample (d) after 1 min deposition. The blank substrate is copper foil. Scale bars are 5 μm. Insets are the zoom-in view with scale bars of 1 μm.

It was also noticed that under mild acidic pH, *hcp* Co forms, while as suggested by Figure 2, *fcc* should be more stable when the nucleation size is small. This then suggests that the size of nuclei may be larger in electrodeposition than in solvothermal reaction. SEM images of samples after 1 min of deposition were taken and shown in Figure 3. It can be seen that only after 1 min of deposition, particles with average sizes of ∼1 μm and 300 nm have formed in N and MA samples, respectively. Samples obtained from even shorter deposition time, such as 10 or 15 s were also examined with SEM. The particle size is not very different from shown in Figure 3, other than that the deposition was much less homogeneous and only isolated island-like areas were covered with deposited particles. The SEM results revealed that electrodeposition in nature is much more inhomogeneous than chemical reactions in solution. Because in solution reaction, nucleation can take place at any arbitrary places in the solution. While in electrodeposition, the nucleation site is limited within the surface area of the electrode. Furthermore, due to the slight inhomogeneity of the substrate in height, shape and electrical resistance, nucleation also prefers to take place in selected sites, instead of the whole surface, resulting island-like features or even dendritic growth. The dendritic growth is, as well-known, very common in metal electrodeposition.^17−20^ Therefore, we speculate that the larger nucleation size pushes the phase boundary between *hcp* and *fcc* phase to much lower pH in electrodeposition.

The effect of kinetics caused by different overpotential was also investigated. When the starting pH was set 1 and overpotential was varied, (1.5 V for HA-LV and 0.8 V for HA-HV) ∼10 times higher current density was resulted in the HA-HV condition than in HA-LV. As a result, despite that the very low pH condition should favor the formation of *fcc* phase, pronounced *hcp* Co forms concurrently together with *fcc* Co upon completion of the electrodeposition, as shown in Figure 1. This is because the driving force of the reaction is large, while the difference in energy of the two polymorphs is relatively small. Therefore, with the large driving force and the fast kinetics (high current density), both phases can nucleate and keep growing.

The morphology of the deposited samples can be seen in the insets of Figure 3. In Figure 3a and 3b, characteristic hexagonal cylindrical rod and hexagonal plate shapes are seen, which is in accordance with the symmetry of the *hcp* phase. In Figure 3c, the HA-LV sample shows much finer particle size and features, likely due to slower and more isotropic growth of the *fcc* phase. In Figure 3d, larger particles and both characteristic shapes of the *fcc* and *hcp* phases can be seen, in agreement with the XRD patterns. It needs to be noted that the *ex situ* XRD and SEM results are taken from particles after growth and ripening, which have much bigger particle sizes than what was calculated in DFT in Figure 2. These results can not be directly used to verify the results of DFT. What should be compared with the DFT results is the early nucleation stage, which calls for in situ investigations.

**Figure 4:**
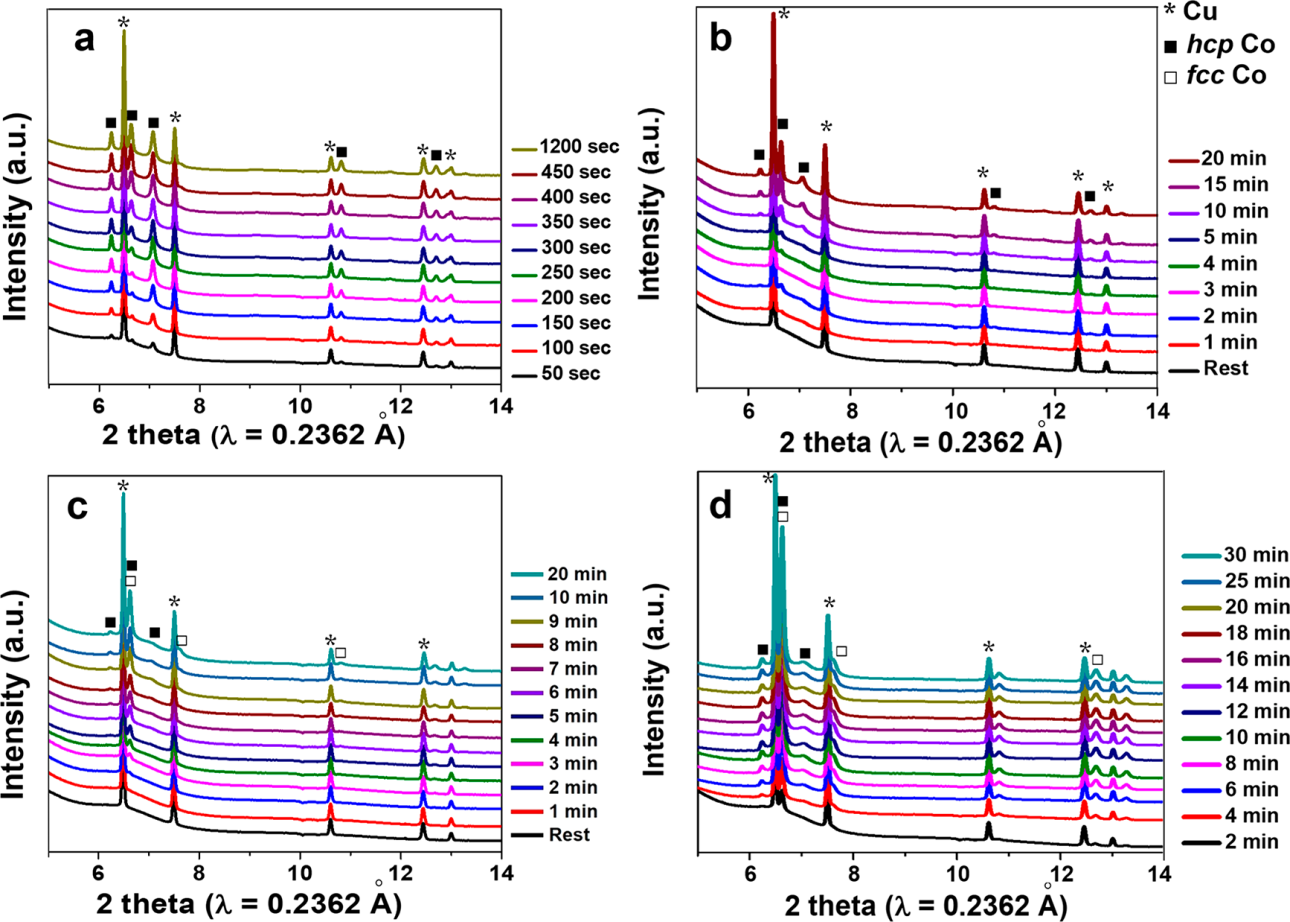
*In situ* XRD of N (a), MA (b), HA-LV (c), and HA-HV (d) samples from nucleation to ripening process.

The *ex situ* XRD results demonstrate the effect of pH and overpotential on phase selection of Co upon completion of phase formation and ripening. To understand the nucleation process, *ex situ* characterization is not sufficient and *in situ* characterization is indeed necessary. While *in situ* Raman/FTIR^21,22^ experiments for electrodeposition have been reported in a number of cases, *in situ* XRD for electrodeposition remains to be very challenging and rarely reported.^23^ It is difficult not only because the existence of electrolyte and the very thin thickness of the deposited layers but also because the deposition reaction is very fast. The deposition can take place in milliseconds once current is applied, which therefore requires high time-resolution. In this work we developed an *in situ* XRD method utilizing the high intensity and flux of synchrotron X-ray beam and the high time resolution of the high speed area detectors. Electrodeposition experiments were conducted with using the same electrolytes and voltages as used in the lab with using the *in situ* cell (Figure S1). XRD signal was collected in real time every 50 or 60 s. Figure 4 shows the *in situ* XRD patterns of N, MA, HA-LV, and HA-HV samples. In Figure 4a, *hcp* phase already forms in the first spectrum, which was collected during the first 50 s of the deposition. While in Figure 4c, the signal of the *fcc* phase can only be observed in the third spectrum, which was taken after 2 min. This comparison confirmed that the nucleation of *fcc* phase is more homogeneous than that for *hcp*. When comparing Figure 4a and 4b, it can be seen that with lower pH, the detectable nucleation of *hcp* phase is slower (after 2 min). Interestingly, the peak intensity and width of the *hcp* phase in the N and MA samples are very different. The intensity of (002) is much higher in sample MA, implying a preferred orientation with the (002) planes perpendicular to substrate. In the *ex situ* XRD patterns shown in Figure 1, it can be also seen the intensity of (002) of MA is lower than that of sample N. The two different relative intensities of the (002) peak actually confirmed the same preferred orientation. Because the *ex situ* XRD was taken in flat plate mode, where the (002) peak is supposed to be lowered, and the *in situ* XRD was taken in transmission mode, where the (002) peak is supposed to be enhanced with such preferred orientation. This observation can also be confirmed by the SEM image in Figure 3b. The hexagonal plates, whose top surface should be (002) facets, are mostly perpendicular to the copper substrate.

**Figure 5:**
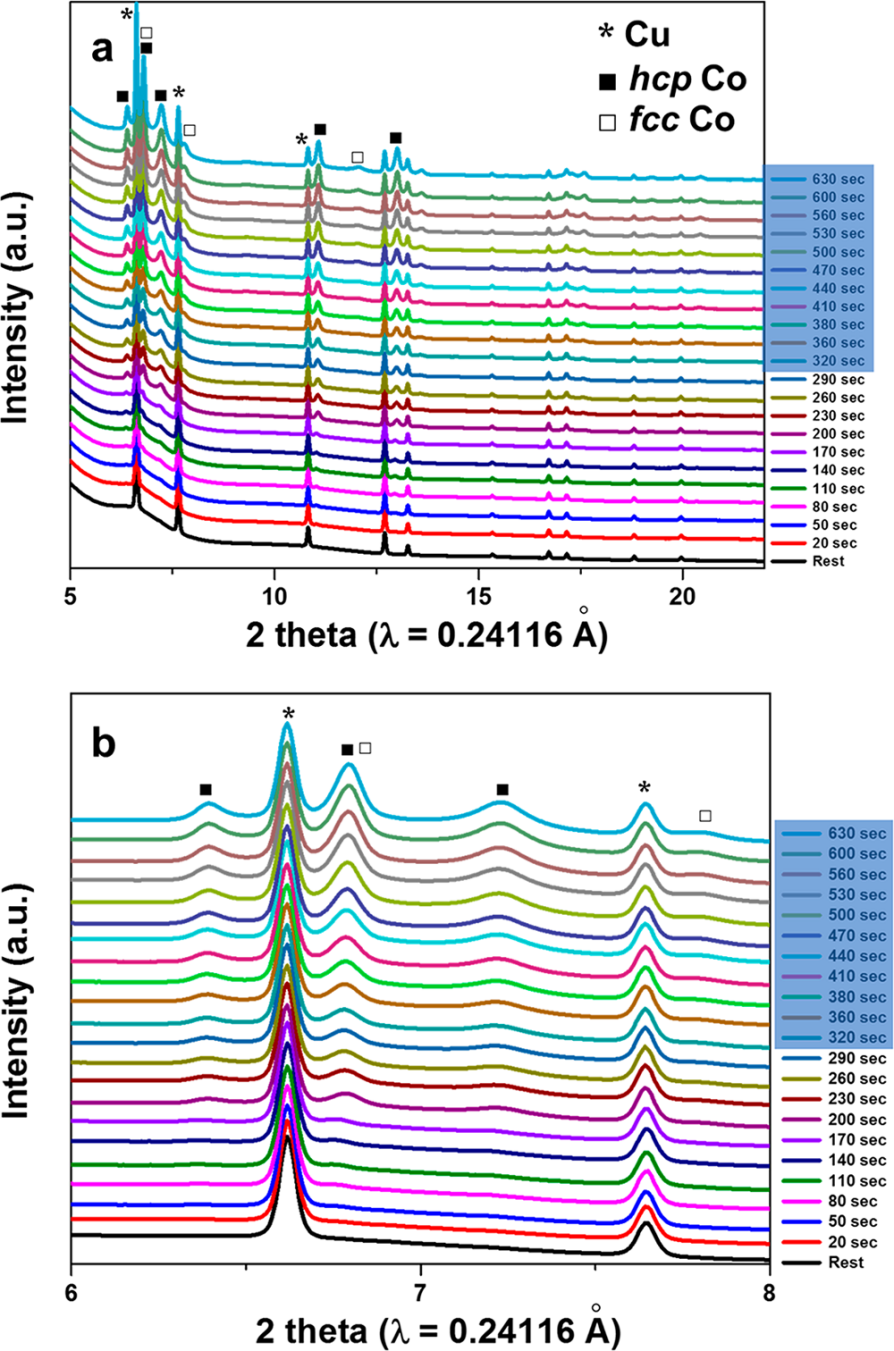
(a) *In situ* XRD pattern of layer-by-layer Co neutral sample. The first 5 min is under low overpotential, while the next 5 min is under high overpotential. (b) The zoom-in of patterns.

The *ex situ* and *in situ* XRD experiment not only reveals the relationship between the phase selectivity and the electrodeposition condition but also offers new opportunities of making new materials and devices with using the insightful knowledge. For example, we conducted layer-by-layer deposition of Co by tuning overpotential with using a constant electrolyte. The deposition started with using low overpotential (current density 10 mA/cm^2^) in a neutral electrolyte. After 5 min of deposition, we tuned the voltage to high overpotential (current density 80 mA/cm^2^). As discussed earlier, the thermodynamic driving force of the reaction is scaled with the overpotential applied. While in our practice, because the dimensions of the cell and the distance between the electrode are fixed and the conductivities of the electrolytes in the experiments are very similar, we use the current density as the scale of the thermodynamic driving force. But it should be noted that when comparing reactions conducted in different electrochemical configurations and setups, overpotential should be used as the scale of the thermodynamic driving force. Figure 5 shows the *in situ* XRD pattern of this two-step process. The XRD patterns show that, as designed, *hcp* Co forms first. After switching to high overpotential (blue-highlighted time labels in Figure 5), *fcc* phase starts to appear. Though the strongest (111) peak of the *fcc* phase largely overlaps with the (002) peak of *hcp*, the faster increasing intensity of these peaks (at ∼6.6°) than that of the other two *hcp* peaks (100) and (101), at 6.2° and 7.1°, respectively, shows the growing of the *hcp* phase, as shown in the zoomed regions in Figure 5b. The formation of this *hcp* → *hcp + fcc* layer-by-layer deposition provides a new means of creating phase controlled double or multilayer thin film structures of Co by just simply tuning the deposition voltage, which may be used for magnetic property or structural property related applications.

In this work, we have demonstrated the phase selection mechanism of Co through electrodeposition by combining *ex situ* and *in situ* XRD characterization. It was found that in close-to-equilibrium deposition conditions, *hcp* Co prefers to form under higher pH, while *fcc* Co prefers to form under lower pH. This finding agrees with previously established nanometric phase diagram of Co and further demonstrates the effectiveness of the nanometric phase diagram. In addition, overpotential is an important factor affecting phase selection in electrodeposition by boosting the kinetics, resulting in codeposition of both stable and metastable polymorphs. Both the thermodynamic and kinetic control can be used to create new phase controlled multilayer Co structures, which can be very useful for many applications.

## Supporting Information Available

Composition of the electrolyte for cobalt electrodeposition, bath, photos and schematic of the in situ XRD setup, and calculated surface energy is available (PDF)
