# Supplementary material for: Understanding the Polymorphism of Cobalt Nanoparticles Formed in Electrodeposition—An In Situ XRD Study
Source: ACS Mater Lett. 2023 Mar 6;5(4):979–84. doi: 10.1021/acsmaterialslett.2c00861 (PMC10074481; doi:10.1021/acsmaterialslett.2c00861)
Supplement: Supplementary file 1 — tz2c00861_si_001.pdf [file tz2c00861_si_001.pdf]

Supplementary Information

Understanding the Polymorphism of Cobalt Nanoparticles Formed in Electrodeposition

—a combined computational and experimental study

Xuetian Ma[1], Yifan Ma[1], Adelaide Nolan[2], Jianming Bai[3], Wenqian Xu[4], Yifei Mo[2], Hailong Chen[1*]

1. Georgia Institute of Technology, the Woodruff School of Mechanical Engineering, 771 Ferst Drive, Atlanta, GA, 30332, USA.

2. University of Maryland, Department of Materials Science and Engineering, 4418 Stadium Drive, College Park, MD, 20742, USA.

3. National Synchrotron Light Source II, Brookhaven National Laboratory, Upton, NY, 11973, USA.

4. Advanced Photon Source, Argonne National Laboratory, 9700 Cass Ave, Lemont, IL, 60439, USA.

[*]Questions should be addressed to: hailong.chen@me.gatech.edu

1. Phase diagram of cobalt

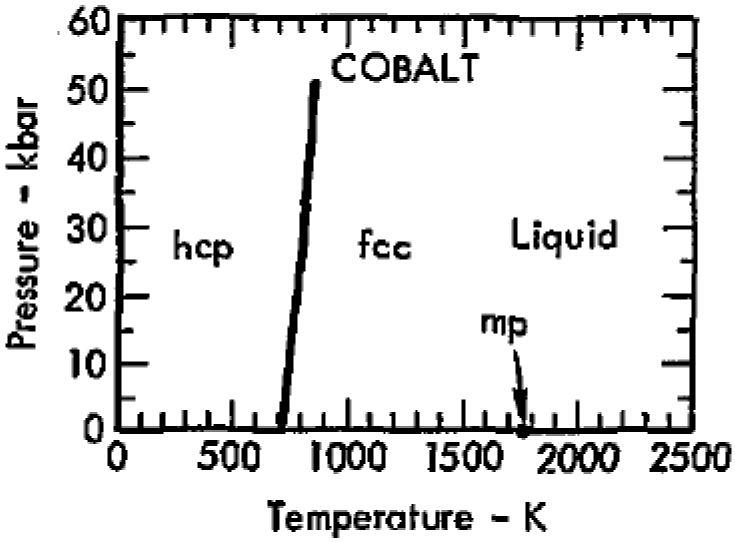

Fig. S1 Bulk phase diagram of cobalt [1]

[1] M. Erbudak, E. Wetli, M. Hochstrasser, D. Pescia, D. Vvedensky, Surface phase transitions during martensitic transformations of single-crystal Co, Physical review letters, 79 (1997) 1893.

2. Chemical composition of cobalt electrodeposition bath*.

Table S1 Synthesis condition of electrodeposited Cobalt under different conditions

Bath Name	*$CoSO_4{\cdot}5H_2O$/M*	*H_3BO_3/M*	*H_2SO_4/M*	*Current density/mA•cm^{-2}*	*Deposition time/min*
N	0.5	-	-	~10	20
MA	0.5	0.1	-	~12	20
HA-LV	0.5	0.1	0.1	~6	20
HA-HV	0.5	0.1	0.1	~65	20

* The solvent of the bath is 20 mL deionized water.

3. *In situ* electrolytic cell assembled on beamline 28-ID-2, National Synchrotron Light Source II.

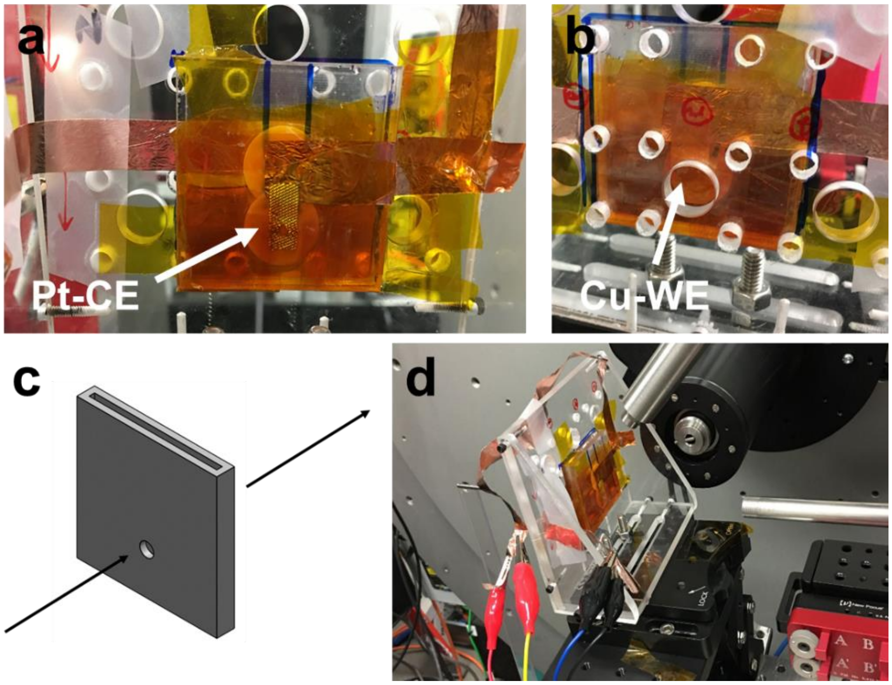

Fig. S2 (a) Front view of electrolytic cell. A platinum mesh with a hole to let beam pass through was used as the counter electrode (CE); (b) Back view of electrolytic cell. A piece of copper foil was used as the working electrode (WE); (c) Schematic view of electrolytic cell. The outer dimension of the cell is 50 mm x 50 mm x 6.35 mm, with thickness of 2.175 mm and diameter of the hole is 5 mm; (d) Overview of final setup on beamline.

4. Relation of average surface energy of various facets of *fcc* and *hcp* Co with respect to different pH conditions.

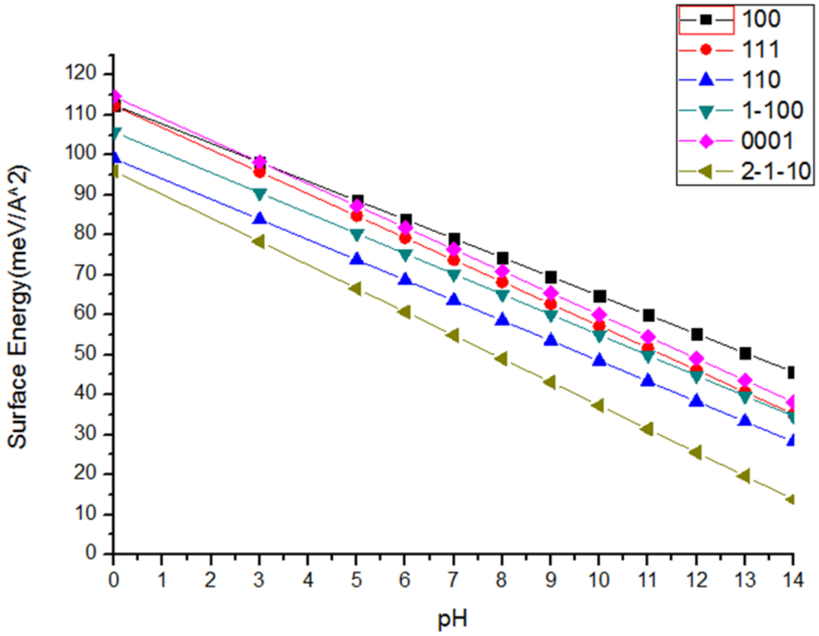

Fig. S3 Surface energy of fcc Co (100) (111) (110) facets and hcp Co (1-100), (0001), and (2-1-10) facets under different pH conditions.
